# In vitro aggregating β-lactamase-polyQ chimeras do not induce toxic effects in an in vivo *Caenorhabditis elegans* model

**DOI:** 10.1186/s12952-017-0080-5

**Published:** 2017-08-22

**Authors:** Roel Van Assche, Charline Borghgraef, Jonathan Vaneyck, Mireille Dumoulin, Liliane Schoofs, Liesbet Temmerman

**Affiliations:** 1 Animal Physiology and Neurobiology, Department of Biology, KU Leuven (University of Leuven), Zoological Institute, Naamsestraat 59, 3000 Leuven, Belgium; 2 Enzymology and Protein Folding, Center for Protein Engineering, InBioS, Institute of Chemistry, University of Liège, Sart-Tilman, 4000 Liège, Belgium

**Keywords:** *Caenorhabditis elegans*, β-lactamase BlaP, Model polyglutamine proteins, polyQ, In vivo protein aggregation, Non-polyQ regions

## Abstract

**Background:**

A series of human diseases are caused by the misfolding and aggregation of specific proteins or peptides into amyloid fibrils; nine of these diseases, referred to as polyglutamine diseases, are associated with proteins carrying an expanded polyglutamine (polyQ) region. While the presence of this latter is thought to be the determinant factor for the development of polyQ diseases, the non-polyQ regions of the host proteins are thought to play a significant modulating role.

**Method:**

In order to better understand the role of non-polyQ regions, the toxic effects of model proteins bearing different polyQ regions (containing up to 79 residues) embedded at two distinct locations within the β-lactamase (BlaP) host enzyme were evaluated in *Caenorhabditis elegans*. This small organism can be advantageous for the validation of in vitro findings, as it provides a multicellular context yet avoids the typical complexity of common studies relying on vertebrate models. Several phenotypic assays were performed in order to screen for potential toxic effects of the different BlaP-polyQ proteins.

**Results:**

Despite the significant in vitro aggregation of BlaP-polyQ proteins with long polyQ regions, none of the BlaP-polyQ chimeras aggregated in the generated transgenic in vivo models.

**Conclusion:**

The absence of a toxic effect of the expression of BlaP-polyQ chimeras may find its cause in biochemical mechanisms present in vivo to cope with protein aggregation (e.g. presence of chaperones) or in *C. elegans’* limitations such as its short lifespan. It is plausible that the aggregation propensities of the different BlaP chimeras containing embedded polyQ sequences are too low in this in vivo environment to permit their aggregation. These experiments emphasize the need for several comparative and in vivo verification studies of biologically relevant in vitro findings, which reveal both the strengths and limitations of widely used model systems.

**Electronic supplementary material:**

The online version of this article (doi:10.1186/s12952-017-0080-5) contains supplementary material, which is available to authorized users.

## Background

The aggregation of proteins or peptides into amyloid fibrils is associated with a series of prevalent and intensively studied neurodegenerative diseases. Amongst these, nine diseases referred to as polyQ diseases - including Huntington’s disease – all arise from an abnormal expansion of an unstable CAG repeat in the coding region of one of the nine associated genes [1]; these expanded CAG repeats are translated into an extended polyglutamine (polyQ) region within the corresponding protein. PolyQ proteins only become pathogenic if they contain a polyQ region longer than a threshold value, situated between 35 to 45 glutamine residues in most of the nine proteins [2]. Moreover, the age of onset of polyQ disorders is inversely correlated with the length of the polyQ region [3], i.e. above the pathogenic threshold, the longer the polyQ region, the earlier the onset [4, 5]. Finally, polyQ regions longer than the pathologic threshold induce the aggregation of the host protein into amyloid fibrils [6, 7]. The exact mechanism of polyQ toxicity is still unknown, but a vast amount of data indicate that protein misfolding and aggregation into amyloid fibrils underlie these processes [1, 8–10].

**Fig. 1:**
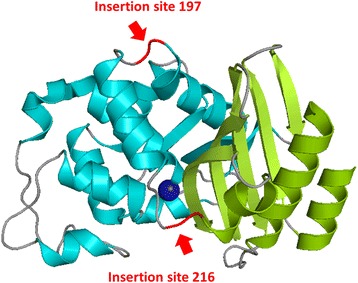
X-ray crystal structure of the β-lactamase from *Bacillus licheniformis* 749/C. The α- and α/β-domains are represented in *light blue* and *light green*, respectively. The two insertion sites 197 (located between helices 8 and 9) and 216 (inserted between helices 9 and 10) are coloured in *red* and are highlighted by two red arrows. The serine active site is represented by a sphere in dark blue. The numbering of the insertion sites, i.e., positions 197 and 216,  refers to the numbering scheme of class A β-lactamases [74],  which correspond to residues 168 and 187 in the sequence of the protein [74]

While the detrimental aggregation propensity of polyQ proteins critically depends on the presence of an extended polyQ region, the properties of the host protein (i.e. the non-polyQ regions) can significantly influence the kinetics of aggregation and the properties of the aggregates formed [1, 11, 12]. Depending on the non-polyQ regions, the aggregation into amyloid fibrils can be favored or prevented [13–16], and the molecular mechanisms of the complex interplay between the ability of expanded polyQ sequences to trigger aggregation and the modulating role of non-polyQ regions is still not fully clarified. For example, the contributions of the sequence, size, topology, structure, stability or dynamics of the host protein are not yet fully addressed. Since most proteins associated with polyQ diseases are rather big and exhibit limited solubility when containing long polyQ sequences [1, 12, 15], a number of artificial model polyQ proteins (i.e. comprising a host protein not associated with any polyQ disease and a polyQ region of different lengths) have been engineered in order to address these unanswered questions [6]. One of these relies on the use of the β-lactamase BlaP (30.4 kDa) from *Bacillus licheniformis* 749/C as host protein [6, 17]. This two-domain host protein (Fig. 1) has been chosen because its thermodynamic and catalytic properties are well known, providing a strong basis to investigate the effects of polyQ insertion [12] but most importantly because two distinct positions (so-called sites 197 and 216, see Fig.1) within the protein have been shown to tolerate amino acid insertions [6, 17]. BlaP is the only host protein that can be produced recombinantly in which long polyQ regions have been successfully inserted at two different locations and whose in vitro properties could be extensively investigated; this is instrumental in assessing how subtle differences between polyQ region insertional environments - without disturbing the overall protein structure - affect the ability of polyQ sequences to trigger aggregation [18]. The aggregation properties of two sets of BlaP-polyQ chimeras with polyQ insertions at either position 197 or 216 recapitulate those of polyQ proteins associated with diseases: there is a threshold polyQ length above which the chimeras form in vitro amyloid fibrils and above the threshold, the longer the polyQ, the faster the aggregation [6, 18]. Moreover, BlaP chimeras bearing a polyQ region at position 216 have a significantly higher aggregation propensity than their counterparts with polyQ insertions at position 197: the polyQ length threshold for fibril formation is lower and above this threshold, chimeras with insertions at position 216 aggregate faster than chimeras with polyQ insertions of similar length at position 197 [6, 18]. This could be because the 197 insertion site is located within the folded α-domain of BlaP, in contrast to the 216 insertion site, which is located at the interface of the α-domain and the α/β-domain. This interdomain insertion is thought to destabilize the interface between the domains and could therefore result in a higher aggregation propensity of BlaP216 chimeras. These results highlight the critical role of subtle modifications in the properties of the non-polyQ region on the in vitro aggregation properties of polyQ proteins.

While artificial proteins with expanded polyQ repeats have been shown to display robust aggregation in vitro, the in vivo effects are unknown. Thus, this study aimed to address this point and to investigate the consequences of subtle differences between polyQ region insertional environments within the same host protein on the in vivo consequences for polyQ protein aggregation. For this, various BlaP-polyQ proteins were expressed in the nematode *Caenorhabditis elegans*. This relatively simple organism was chosen because it has become a popular model for studying development, aging, neurobiology and protein aggregation, amongst others [19, 20]. Its transparency, fast reproduction, short lifespan, easy phenotyping, powerful genetic toolbox and fully sequenced genome are some of its widely appreciated advantages [21]. Additionally, in *C. elegans*, evidence has been gathered showing that protein quality control processes and protein degradation pathways influence toxic protein aggregation [22, 23]. Many protein-misfolding disorders have already been modelled with success in this animal, as supported by the availability of diverse amyloid-β, tau, superoxide dismutase and polyQ expressing strains [24–27]. This nematode has also proved its worth in compound screens against toxic protein aggregates [28].

Specifically with regards to modelling polyQ disorders, several efforts relying on *C. elegans* as an in vivo context have already been made. Most of these models exhibit a C-terminal polyQ region. For instance, muscle-specific expression of terminal polyQ regions fused to only 17 amino acids from the dentatorubural pallidoluysian atrophy protein were employed to study the protective effects of *C. elegans* p97 homologs [29]. In addition, several models rely on fusions of polyQ regions with fluorescent proteins, allowing direct observation of in vivo aggregation thanks to *C. elegans’* transparency. While neuronal expression of such proteins certainly has been used (e.g. [25]), body wall muscle expression of polyQ fusion proteins is popular in *C. elegans* [30–32], the latter having the advantage of mobility impairment as an easy readout. These studies also allowed the identification of several genes whose reduced expression enhanced the observed aggregation, and contributed to our understanding of the role of protein homeostasis in polyQ diseases.

Also in *C. elegans*, a number of studies to date have already focused on the effect of polyQ regions embedded within the host protein. For example, several studies based on N-terminal fragments of huntingtin delivered insights into the correlation between polyQ region length and the severity of the observed defects in *C. elegans* [33], neuronal toxicity of [34], and ubiquilin protection against [35] polyQ-containing huntingtin fragments, and the age-associated remodelling of neurons [36]. In another study using polyQ regions longer than 60 repeats and embedded within ataxin-3, an imbalance in cellular aggregation-associated proteotoxicity was observed [37].

In general, phenotypic readouts are observed for polyQ regions longer than a certain threshold length, however, its value varies significantly (from 40 Q to >120 Q) over different studies. This observation holds true in worms, yeast, flies and mice [34, 38]; and the observed threshold is often larger than that observed in human diseases [34]. Knowing this, it is however difficult to draw any conclusions regarding the specific role of the non-polyQ regions, which differ between these studies, since many other parameters – such as the spatiotemporal expression or the age of the animals – vary over studies as well. Still, there is evidence to clearly identify the non-polyQ regions as modulators of in vivo polyQ aggregation. For example, full-length and truncated human ataxin-3, both containing polyQ regions of various sizes were expressed in the nervous system *of C. elegans*, which showed that the truncated version of the protein aggregates faster than the full length protein [39]. Another study relied on transient cellular expression of GFP fusion proteins containing 56 Qs and a fixed number of flanking amino acids (8 N-terminal and 9 C-terminal). These sequences were identical to those known to also flank the polyQ region in various proteins associated with polyQ diseases, and the study showed that these significantly modulate the aggregating properties of the polyQ-GFP fusion proteins [40].

In line with these studies, our work aimed to expand on the knowledge regarding in vivo effects of protein context. By expressing, *in C. elegans*, various BlaP-polyQ chimeras in which a polyQ region is inserted at different locations, we aimed to evaluate whether the observed effects of the location of the polyQ insertion on the in vitro aggregating properties would also be observed in vivo; or in other words whether the in vivo aggregation would be as sensible to subtle changes in the polyQ environment as the in vitro aggregation is.

## Methods

### Strains and culturing

Strains (N2 wild type, SJ17 (*xbp-1(zc12)*) III, CL4176 *dvIs27* [*pAF29 (Pmyo-3::A*β*(1-42)::let-383 3’UTR) + pRF4 (rol-6(su1006))] X*, CL2120 *dvIs14* [*pCL26 (Punc-54/A*β*1-42) + pCL12 (mtl-2::GFP)]*, AM 140 *rmIs132* [*Punc-54::Q35::YFP*]) used in this article were obtained from the *Caenorhabditis* Genetics Center (CGC) (University of Minnesota). Plasmids for *Punc54::Q82::YFP, PF25B3.3::Q0::CFP and PF25B3.3::Q86::CFP* expression were a kind gift of the Morimoto lab [32, 41]. Strains were cultured on standard nematode growth medium (NGM) seeded with *Escherichia coli* OP50 [42] and all experiments were conducted at 20 °C. To generate age-synchronized worm populations, adult animals were transferred to fresh NGM plates, allowed to lay eggs for 2 h and thereafter removed. The offspring was then used in the experiment.

### Construction of transgenic *C. elegans* strains expressing BlaP-polyQ chimeras

A series of transgenic *C. elegans* expressing different BlaP-polyQ chimeras (BlaP197Q0, BlaP197Q58, BlaP197Q72, BlaP216Q0, BlaP216Q55, BlaP216Q79, under the control of different promoters were created (Table 1). All of the BlaP-polyQ chimeras were expressed under the control of the body wall muscle-specific *unc-54* promoter. BlaP216Q0, BlaP216Q55 and BlaP216Q79 were additionally expressed under control of the constitutive and ubiquitous *rpl-28* promoter. BlaP197Q72 was also expressed in an AM140 background, expressing Q35::YFP in body wall muscle cells. BlaP197Q0 and BlaP216Q0 refer to BlaP chimeras where a PG dipeptide has been introduced at position 197 and 216. This di-peptide originates from the addition of the SmaI restriction site at the genetic level to allow further polyQ region insertion [6].

**Table 1:** List of generated transgenic strains

Promoter	Background strain	Location of polyQ insertion	PolyQ length	Protein name
Body wall muscle cells	N2	197	0	BlaP197Q0
Body wall muscle cells	N2	197	58	BlaP197Q58
Body wall muscle cells	N2	197	72	BlaP197Q72
Body wall muscle cells	N2	216	0	BlaP216Q0
Body wall muscle cells	N2	216	55	BlaP216Q55
Body wall muscle cells	N2	216	79	BlaP216Q79
Ubiquitous	N2	216	0	BlaP216Q0
Ubiquitous	N2	216	55	BlaP216Q55
Ubiquitous	N2	216	79	BlaP216Q79
Body wall muscle cells	AM140	197	72	BlaP197Q72

Promoter, background strain, location of insertion, length of polyQ region and protein name are indicated

Sequences coding for the BlaP chimeras with insertions at position 197 (i.e. BlaP197 chimeras) were amplified from plasmid pNY (i.e. from pNY-BlaP197Q0, pNY-BlaP197Q55 and pNY-BlaP197Q79, [7]) using 5′-ACACACGCTAGCACGGAGATGAAAGATGATTT-3′ and 5′- CTGCTGTAGCTCGTGGTGGTGGTGGTGGGGCCCT-3′ primers. These sequences were inserted between unique NheI and SacI sites of the pPD30.38 vector (Addgene). Sequences coding for the BlaP chimeras with insertions at position 216 (i.e. BlaP216 chimeras) were inserted in pPD30.38 and L4455 vectors at the unique SacI site using Gibson Assembly® Master Mix (New England Biolabs). Sequences coding for these chimeras were amplified from a pET28b (pET28b-BlaP216Q0, pET28b-BlaP216Q55 and pET28b-BlaP216Q79), vector template using 5′- ATGGTATTGATATCTGAGCTATGAAAGATGATTTTGCTAAACTG-3′ and 5′- ATGACAGCGGCCGATGCGGAGCTTTTCCACGTACGTTG-3′ primers for later insertion into pPD30.38, and 5′- AAATATCCGACGCTCTCGTGATGAAAGATGATTTTGCTAAACTG-3′ and 5′- ATTTTTTCTGAGCCAATCCCGGGTTTCCACGTACGACGTTG-3′ primers for later insertion into L4455.

For the BlaP197 chimera the sequencing revealed that the polyQ region had a length slightly different than in the parent vector (i.e. 58Q instead of 55Q, and 72Q instead of 79Q). Therefore, these two chimeric proteins do contain a polyQ region whose length is slightly different than that of the proteins used to study in vitro aggregation [6, 18].

All constructs were microinjected at high concentration (70 ng/μl) into the gonads of young adults, together with co-injection marker *Pelt-2::gfp* (50 ng/μl) and 1 kb generuler DNA ladder (Thermo Scientific) as carrier DNA (17 ng/μl). An extrachromosomal array is formed and is transmitted to the offspring, eventually generating multiple stable transgenic strains.

### Western blot analysis

The expression of BlaP chimeras by the worms was investigated by Western blot analysis under denaturing conditions. 50 synchronized adult worms were picked in 30 μl S-buffer [42]. Subsequently, worms were centrifuged (800 x g, 3 min, 4 °C) and 15 μl buffer were removed. Next, 15 μl of 2-mercaptoethanol enriched Laemmli buffer (2×) (1,610,737, BioRad) were added to the worm pellet and the samples were incubated for 15 min at 70 °C; during this process, the samples were vortexed every 5 min. Samples were stored at −80 °C until further use.

Proteins were separated on a precast SDS/PAGE gel (4-12% Midi gels, Biorad), for this purpose 15 μl of each sample were loaded on the gel. After separation, the proteins were blotted on a polyvinylidene difluoride membrane (Biorad) and a coomassie-based total protein stain was performed (Additional file 1: Figure S1). A blocking step (2 h) was conducted using 5% blocking agent (GE Healthcare). The membrane was incubated overnight at room temperature with primary mouse anti-polyQ antibody (1/1000 dilution in Tris-Saline pH 7.6, 5TF1-1C2, Millipore) or primary rabbit anti-GFP antibody (1/1000 dilution in Tris-Saline pH 7.6, ab6556, Abcam). Horse Radish Peroxidase-conjugated rabbit anti-mouse (1/50000 dilution in Tris-Saline pH 7.6, P0161, Dako) and anti-rabbit antibodies (1/50000 dilution, P0448, Dako) were used as secondary antibodies for visualization with Supersignal West Dura (Thermo Scientific).

### BlaP enzymatic assay

A native protein extraction was performed as follows: 50 worms were collected from NGM plates with S-buffer and washed 3 times. Worms were suspended in native protein extraction buffer (50 mM Tris-HCl, pH 7.5, 0.1 mM EDTA, 1 mM β-mercaptoethanol +1 tablet of a protease inhibitor cocktail for 50 ml buffer (04693116001, Roche diagnostics), in MagNa Lyser Green Beads tubes (Roche). All samples were homogenized (MagNa Lyser, Roche using 3 cycles of 10s at 4800 rpm, samples were put on ice for 5 min in between the cycles) and centrifuged for 20 min at 16000 *g* and 4 °C. Supernatant was transferred to a new tube and kept at −80 °C until further use.

A qualitative enzymatic test was carried out to detect the presence of functional BlaP in the worm extracts by mixing 15 μL of the extract with 0.1 mM nitrocefin solution in 50 mM phosphate buffer pH 7; in the presence of functional BlaP nitrocefin turns red. For the BlaP197Q72 strain, quantitative measurements were carried out by measuring the initial rate of hydrolysis of nitrocefin (95 μM in 50 mM phosphate buffer pH 7) recorded at 486 nm, for 120-180 min at 25 °C using a Tecan plate reader Infinite 200. The initial rate of hydrolysis (ΔA.min^−1^) is given by the slope of the graph representing the absorbance as a function of time when less than 10% of substrate is hydrolyzed. Initial rate of hydrolysis of nitrocefin obtained with known concentrations of BlaP197Q79 were also measured to draw standard curves from which the concentration of functional BlaP197Q72 chimera in worm extracts could be derived. At least triplicate measurements were carried out for each worm extract and standard solutions, and at least two independent worm extracts were prepared. In order to ensure that the enzyme was not degrading in the worm extract, pure BlaP197Q79 was incubated in the worm extracts for 60 min and then the initial rate of hydrolysis of cephalothin (90 μM in 50 mM phosphate buffer pH 7) was compared to that of the same enzyme incubated at the same concentration in phosphate buffer pH 7 for one hour.

### Locomotion assay

Locomotion of transgenic animals was determined by recording the average speed of different transgenic strains using unseeded NGM plates (i.e. in absence of food). Worms (*n* = 12-24) were recorded for 1 min using a ToupCam (Touptek Photonics, China) equipped on an M165 FC microscope (Leica, Germany). Movies were inverted creating a white background/black worm format for further analysis using the Parallel Worm Tracker [43]. Statistical analysis was performed using one-way ANOVA (Graphpad Prism 5, GraphPad Software, USA). *P* values <0.05 were considered significant.

### Dot blot analysis

The relative expression levels of some BlaP-polyQ chimeras and of Q82::YFP animals were confirmed by dot blot. For this purpose, worm extracts were prepared and collected as described before (Western blot analysis).

2 μl of each sample was spotted on a nitrocellulose membrane and the samples were allowed to dry for 1 h. The membrane was then incubated at room temperature (1) for 2 h in 5% blocking agent (GE Healthcare) and (2) with primary mouse anti-polyQ antibody (1/1000 dilution in Tris-Saline pH 7.6, 5TF1-1C2, Millipore) or primary rabbit anti-Histone H3 antibody (1/20000 dilution in Tris-Saline pH 7.6, ab8580, Abcam). Horse Radish Peroxidase-conjugated rabbit anti-mouse (1/50000 dilution in Tris-Saline pH 7.6, P0161, Dako) or goat anti-rabbit (1/50000 dilution in Tris Saline, pH 7.6, P0160, Dako) were used as secondary antibodies for visualization with Supersignal West Dura (Thermo Scientific).

Dot blot signals were analysed using ImageJ. Upon removal of background signals, the polyQ signal was normalized to the Histone H3 signal, which functioned as an endogenous loading control.

## Results

### Qualitative analyses show that *C. elegans* expresses BlaP-polyQ chimeras

**Fig. 2:**
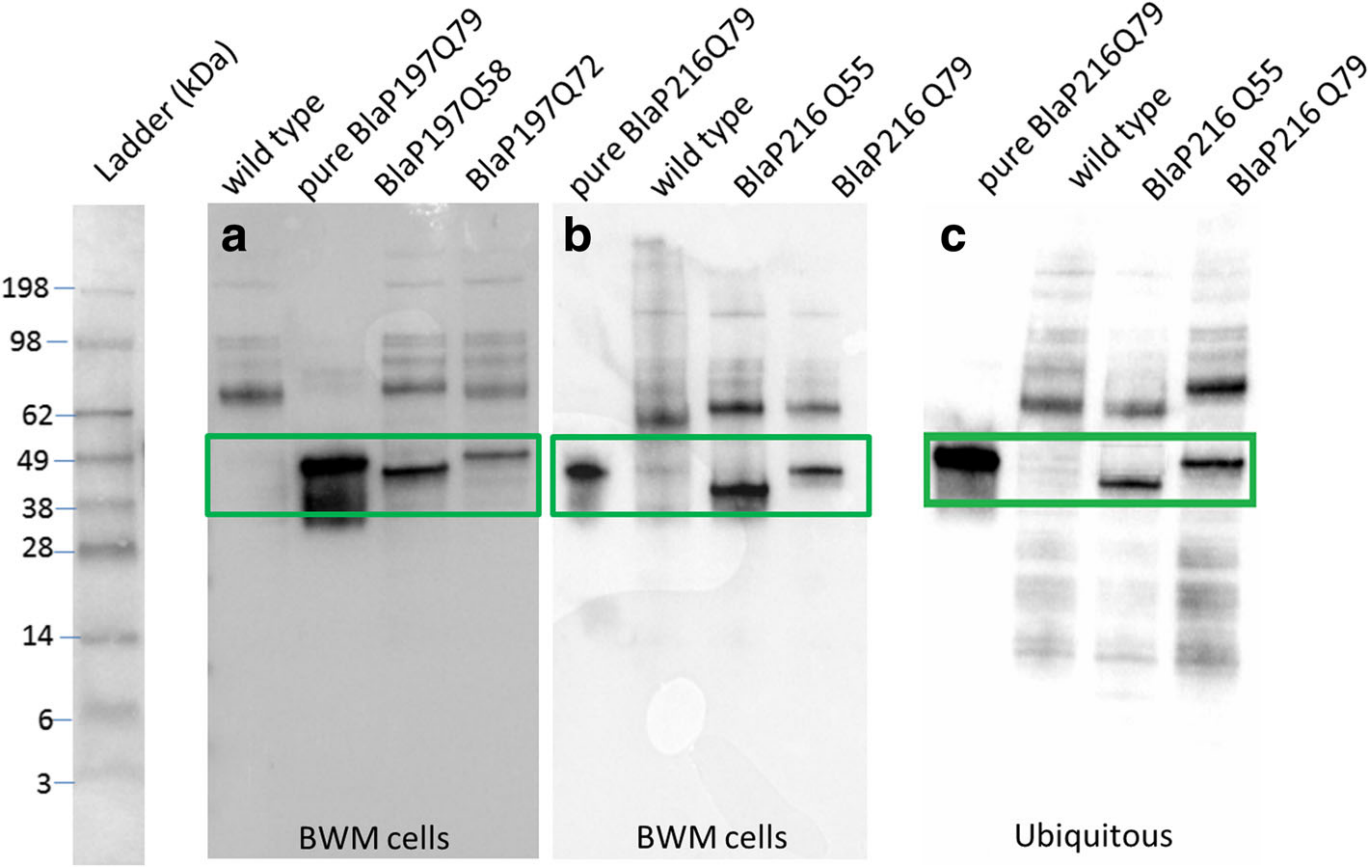
Verification of the expression of BlaP197- and BlaP216-polyQ chimeras using Western blot with a primary antibody against polyQ. **a** Transgenic strains expressing BlaP197Q58 and BlaP197Q72 in body wall muscle (BWM) cells (*Punc-54*). **b** Transgenic strains expressing BlaP216Q55 and BlaP216Q79 in the BWM cells, or (**c**) in all cells (*Prpl-28*). One μg pure BlaP197Q79 or BlaP216Q79 was added as a positive control and protein extracts from wild-type worms function as a negative control

Transgenic strains expressing BlaP197Q0/58/72 and BlaP216Q0/55/79 in the body wall muscle cells, and ubiquitously expressing BlaP216Q55/79, were generated. The expression of BlaP-polyQ chimeras under the control of either the *Punc-54* muscle-specific promotor or the *Prpl-28* ribosomal promotor was confirmed using Western blot analysis (Fig. 2). A band corresponding to the expected molecular weight is observed in extracts of worms expressing BlaP197Q58, BlaP197Q72, BlaP216Q55 or BlaP216Q79. Wild type worms, as is to be expected, do not express BlaP.

**Fig. 3:**
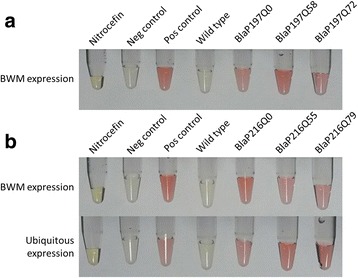
**a**) Nitrocefin assay of transgenic strains expressing A) BlaP197Q0/58/72 in the body wall muscle cells (BWM) and **b)** BlaP216Q0/55/79 in body wall muscle cells or ubiquitously supports functional BlaP expression. For each series, the first tubes represent the endogenous colour of nitrocefin before BlaP hydrolysis. Next, a negative control (extraction buffer), positive control (pure BlaP197Q79 and BlaP216Q79), extracts from wild-type worms and the transgenic BlaP strains are shown. Pictures were taken immediately after addition of the substrate, data are qualitative and show that all strains express functional β lactamase

The presence of functional chimeric proteins produced in *C. elegans* was further demonstrated using the enzymatic activity of BlaP as readout (Fig. 3). The results confirmed that indeed, the native forms of BlaP197Q72 and BlaP216Q79 are present in our transgenic strains (Fig. 3).

### Expression of BlaP-polyQ does not affect *C. elegans* locomotion or unfolded protein response

**Fig. 4:**
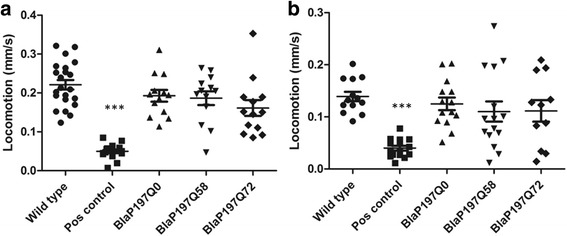
Overall spontaneous speed of locomotion of transgenic BlaP197-polyQ chimera strains on (**a**) day 1 and (**b**) day 3 of adulthood is similar to wild type speed. Wild type animals and a slowly moving amyloid-Aβ42 expressing strain serve as appropriate normal (negative) and slow (positive) controls, respectively (*n* = 13-22 for each condition). The values obtained for the positive control strain are significantly different from those obtained for the wild-type animals at both times [*P* values <0.001 (***)]. Error bars indicate standard error of the mean

Toxic protein aggregation in the muscle cells of the worm will lead to impaired locomotion [32, 44–46], which provides a robust readout for polyQ proteotoxicity in an in vivo system. We evaluated overall locomotion of the BlaP197-polyQ chimera strains on days 1 and 3 of adulthood (Fig. 4). Unlike the slow positive control (amyloid-β expression in *C. elegans* body wall muscle cells is known to affect locomotion [47]), transgenic BlaP197-polyQ chimera strains display normal locomotion at these early days of adulthood. As observed in previous research [48] and being a result of aging, the overall speed of the worms is lower at day 3 of adulthood as compared to day 1 adult worms (Fig. 4).

**Fig. 5:**
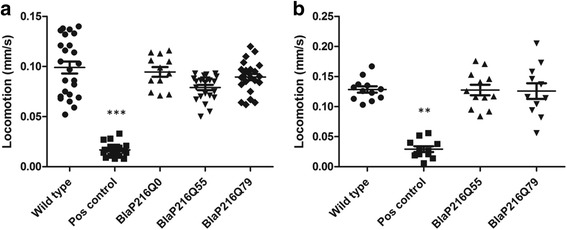
Overall locomotion of transgenic strains with (**a**) body wall muscle cells expression (**b**) and ubiquitous expression of BlaP216Qx on day 3 of adulthood. Wild type animals and a slowly moving amyloid-β expressing strain serve as appropriate normal (negative) and slow (positive) controls (*n* = 12-24 for each condition). The values obtained for the positive control strain are significantly different from those obtained for the wild-type animals at both times [*P* values <0.001 (***) in (A) and <0.01 (**) in (B)]. Error bars indicate standard error of the mean

Based on in vitro data [18], it is to be expected that BlaP216-polyQ chimeras are more aggregation-prone than their BlaP197-chimera counterparts. Therefore, they may have a more pronounced impact on *C. elegans* locomotion. The expression of BlaP216Q55 or BlaP216Q79, be it ubiquitous or in the body wall muscle cells, did however not result in an altered locomotion phenotype (Fig. 5).

**Fig. 6:**
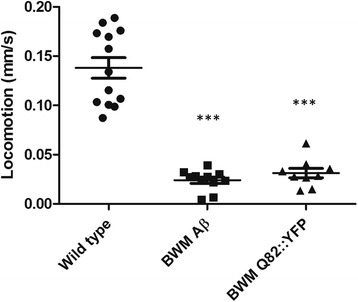
Positive control strains expressing either Aβ- or Q82:YFP display similar reductions in locomotion as compared to wild-type animals. Data collected on day 3 of adulthood. (*n* = 9-13 for each condition). Transgenic strains expressing either Aβ or Q82::YFP displayed a significant locomotion defect (both *P* values <0.001 (***)). Error bars indicate standard error of the mean. BMW: body wall muscle cells specific expression

The positive control strain used in the above-mentioned locomotion assays expresses Aβ42 in the body wall muscle cells of *C. elegans* and exhibits a consistent slow phenotype [23], ensuring proper execution of the experiment. To prove that a similar observation can indeed be used for estimating polyQ toxicity, a strain expressing 82 Qs fused to the N-terminus of YFP (Q82::YFP) in the body wall muscle cells of *C. elegans* was also subjected to the assay (Fig. 6). This strain is characterized by fast and strong protein aggregation [32], demonstrating that the observed locomotion of animals expressing aggregating polyQ proteins is indeed altered to the same extent as that of worms expressing Aβ (Fig. 6).

Since unfolded protein response (UPR) levels are known to be elevated as a result of protein misfolding/aggregation [49], a tunicamycin UPR stress assay was conducted to serve as a potential alternative readout. Unlike the positive control (*xbp-1* mutant) which is unable to induce an unfolded protein response [50], Q82::YFP and transgenic BlaP-polyQ strains show no increased mortality due to the tunicamycin-induced UPR stress (Additional file 1: Figure S2).

As locomotion-based results indicate the absence of polyQ toxicity, hence of aggregates in our strains, we also tried to visualize and quantify potential aggregates by means of both a thioflavin stain and SDD-AGE analyses (Additional file 1: Figure. S3-5). We however faced technical limitations and rely on the indirect readout of normal locomotion (Figs. 4, 5 and 6) to suggest that BlaP-polyQ chimeras do not significantly aggregate in our strains.

### BlaP-polyQ expression levels in non-aggregating strains are similar to polyQ expression in positive controls

While no clear functional readout could be observed, it is still possible that some BlaP-polyQ chimeras aggregate in these animals, yet not in sufficient amount to bring about functional consequences. One obvious explanation for the absence of significant aggregation or phenotypic effects would be an insufficient level of transgenic protein expression. Based on sample availability, relative and absolute expression levels for several strains were evaluated using Western blot, dot blot and enzymatic activity analyses.

**Fig. 7:**
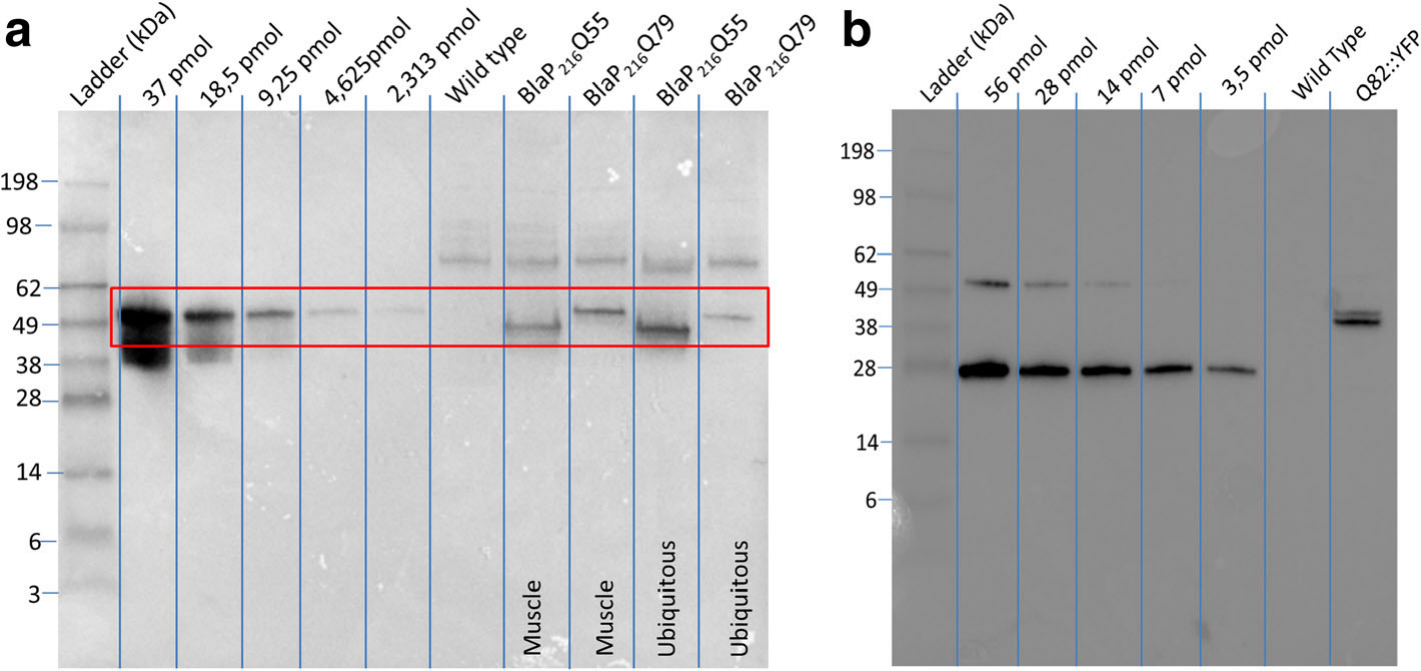
Expression levels of (**a**) BlaP-polyQ chimeras and (**b**) 82Q::YFP in transgenic strains expressing BlaP216Q55/79 and Q82::YFP in the body wall muscle cells. Twofold dilution series of pure BlaP197Q79 and YFP were analyzed in order to estimate transgenic expression levels using Western blot (mouse anti-polyQ antibody, 5TF1-1C2, Millipore). Additional bands in the (B) dilution series are observed, representing dimers of pure YFP. The Q82::YFP signal is observed at 38 kDa due to the additional weight of the polyQ region. *Vertical lines* were added to the image to aid in lane discrimination of the single immunoblot represented in the figure

The Western blot signal of BlaP-polyQ in the transgenic strains was compared to a dilution series of pure BlaP-polyQ chimeras. Concentrations ranging around 20 to 60 μM were estimated inside a single worm, assuming cylindrical adults of 1 mm in length and 40 μm in diameter, and a homogenous distribution of the protein throughout the animal (Fig. 7a). Using similar assumptions, levels of the clearly aggregating Q82::YFP - under control of the same body wall muscle cell promoter as the BlaP216Q55/79 strains - were estimated to be around 20 μM (Fig. 7b). This indicates that BlaP chimeras do not aggregate even though they are expressed at similar concentrations as Q82::YFP in *C. elegans*.

**Fig. 8:**
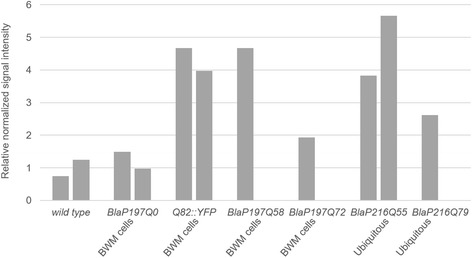
Relative PolyQ protein levels as determined by dot blot analysis. Total polyQ protein levels of transgenic strains expressing BlaP197Q0, BlaP197Q58 and BlaP197Q72 in body wall muscle (BWM) cells (*Punc-54*), or ubiquitously expressed BlaP216Q55 and BlaP216Q79 (*Prpl-28*) were determined by dot blot analysis using an anti-polyQ antibody. Pure BlaP197Q79 or BlaP216Q79, as well as a transgenic strain expressing Q82::YFP in BWM were added as positive controls; protein extracts from wild-type worms function as a negative control. The signal was analysed using ImageJ. After background removal, the polyQ signal was normalized to the histone H3 signal. Each *bar* represents an individual biologically independent sample. The average of both wild type values was set to 1

We in addition relied on dot blot analyses (Fig. 8) of some of these strains to compare the relative level of expression of BlaP-polyQ chimeras to that of Q82::YFP. The results obtained confirmed that the expression levels of the BlaP-polyQ chimeras are comparable to that of Q82::YFP.

Finally, we aimed to confirm expression estimates via enzymatic activity measurements. Pure BlaP197Q79 was used to establish a standard curve from which the concentration of the protein in the worms could be derived (Table 2). In line with expectations and under the same approximations of cylindrical worm volume and homogenous protein distribution, this methodology estimated BlaP197Q72 concentration to be 33.9 ± 6.2 μM in the worm. This confirmed that functional expression levels of this chimera are within the same range as the total concentrations calculated above (Fig. 7).

**Table 2:** Enzymatic activity measurements

Sample	Volume	Initial rate of Hydrolysis (ΔA/min)			[BlaP197Q79] in the extract (nM)			Average [BlaP197Q79] in the extract (nM)	Average concentration in 50 worms (~0.063 μL)
Replicate		1	2	3	1	2	3		
BlaP197Q72 extract 1	330 μL	0.0056	0.0053	0.0056	5.71	5.46	5.73	5.63 ± 0.15	29.5 ± 0.8 μM
BlaP197Q72 extract 2	365 μL	0.0071	0.0062	0.0061	7.26	6.30	6.21	6.59 ± 0.58	38.2 ± 3.4 μM

The initial hydrolysis rate of nitrocefin is given by ΔA.min^−1^, the slope of the graph representing the absorbance as a function of time when less than 10% of substrate is hydrolysed

Based on the enzymatic activity measurements and assumptions detailed in the main text, an average concentration of 33.9 ± 6.2 μM BlaP197Q79 could be calculated

### Seeding polyQ aggregation does not induce aggregation of BlaP197Q72

**Fig. 9:**
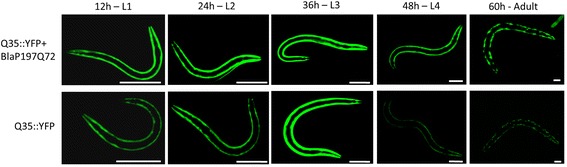
Expression of BlaP197Q72 in a pro-aggregating Q35::YFP background does not result in a faster aggregation process. Expression of BlaP197Q72 in a pro-aggregating Q35::YFP background was established and every 12 h - starting from the L1 stage and until adulthood - the aggregation pattern was visualized. The upper panels represent the transgenic strain expressing BlaP197Q72 in a pro-aggregating Q35::YFP background, the *lower panels* represent the transgenic Q35::YFP strain as a control. *Scale bar* represents 0.1 mm

Since no clear phenotypic defect or protein aggregation were observed in the BlaP transgenic strains, it can be asked whether the timescale needed for in vivo aggregation might be drastically longer than that of the in vitro process. In order to speed up the in vivo aggregation of the BlaP chimeras in our model system, a seeding assay was performed. By expressing BlaP197Q72, of which the concentrations were in the expected order of magnitude, in a pro-aggregating genetic background, facilitated aggregation can be expected [20]. The AM140 strain expresses a Q35 region fused to YFP in its body wall muscle cells. Aggregation can be inferred from the appearance of fluorescent spots in these cells, replacing the normal, diffuse YFP signal [32]. By expressing the aggregation-prone BlaP197Q72 chimera in this background, a faster aggregation process might be expected. However, we did not observe such accelerated aggregation for BlaP197Q72 (Fig. 9).

## Discussion

Protein aggregation is associated with several neurodegenerative disorders [51–54]. An impaired protein homeostasis greatly impacts the aggregation process, but the mechanisms behind this principle have not yet been fully elucidated [55]. Because numerous biochemical events influence protein homeostasis, in vivo analysis of protein aggregation is indispensable to investigate potential toxic effects of in vitro aggregation-prone proteins and to investigate how much these latter depend on the nature of the protein that aggregates within an in vivo context.

Different in vitro aggregation-prone BlaP197- and BlaP216-polyQ chimeras were functionally expressed in the body wall muscle cells of *C. elegans* (Figs. 2 and 3), a tissue chosen because of its easy phenotypic readout upon toxic protein aggregation [32, 35, 56, 57]. In addition, the theoretically more toxic BlaP216Q79 chimeras were also expressed ubiquitously, an intervention that should generate a most severe phenotype. However, we never observed any clear aberrant locomotion phenotype, despite proper expression of the BlaP-polyQ chimeras in all strains and the clear presence of enzymatic activity (Figs. 2, 3, 4, 5 and 6). This suggests that aggregation of BlaP chimeras may not be taking place in these models.

In vitro aggregation studies show that under native conditions (i.e. in 50 Mm phosphate buffer, 150 mMNaCl, pH 7 at 25 or 37°c and protein concentration of 40 μM) BlaP197Q79, BlaP216Q55 and BlaP216Q79 readily form fibrillar aggregates [6, 17, 18, 58]. Because the transgenic strains expressing these proteins do not show significant aggregate formation, it can be asked how *C. elegans* copes with these otherwise aggregation-prone entities. Such questions have also been raised in other organisms. *C. albicans* seems to exhibit mechanisms allowing this organism to properly cope with polyQ aggregates without causing any toxic effects [59]. Whereas *C. elegans* data are yet inconclusive, this worm also seems to cope better with several stress-inducing factors, suggesting a more effective proteostasis. Additionally, Brignull et al. indicated that in *C. elegans*, protein quality control mechanisms and capacity can vary widely among neurons implying discrepancies depending on the cells used to model protein aggregation [41]. Additionally, an asymmetric inheritance of damaged proteins is observed in *Drosophila melanogaster, Escherichia coli, Saccharomyces cerevisiae* and mouse or human stem cells*.* As such, daughter cells tend to be more protected from toxic effects of damaged proteins. For example, stem cells expressing polyQ aggregates of ataxin-3 are believed to employ a specific mechanism to remove damaged proteins, which would explain the absence of large aggregates in these cells [60–62]. Moreover, according to the supersaturation hypothesis of aggregation-prone proteins [63]*,* the level of expression is crucial. In vitro aggregation kinetics of BlaP-polyQ chimeras have been described using relatively high concentrations of BlaP (≥ 40 μM, Scarafone et al. 2012; unpublished results). The estimated amount of BlaP expressed in *C. elegans* is nevertheless somewhat in that range as shown by Western blot, dot-blot (Figs. 7 and 8) and activity measurements (Table 2). It should be noted that our non-integrated transgenic *C. elegans* strains, as can be expected, are characterized by a range of transgene expression levels, somewhat impeding highly controlled experiments. Since - even when taking such variation into account - these levels of expression are also similar to those detected in well-established aggregating polyQ strains (Figs. 7.b and 8), the aggregating propensities of BlaP are probably too low to cause a defined in vivo effect.

In contrast to in vitro studies, protein quality control processes provided by chaperones, ubiquitin-proteasome and autophagy-lysosome degradation systems all act against the aggregation process in vivo. Although one might assume that the UPR is elevated in the transgenic strains due to the expression of different BlaP-polyQ chimera proteins, no increased sensitivity to UPR stress was observed in the tunicamycin UPR stress assay (Additional file 1: Figure S2). *C. elegans* might cope in a different way with overexpression of potentially toxic proteins since Q82::YFP expression did also not result in an increased sensitivity to UPR stress.

The conformational state of the BlaP moiety influences the formation and elongation of amyloid fibrils in vitro [18] and fibril formation of some BlaP-polyQ chimeras takes only a few hours in vitro [6]. Therefore, next to the in vivo context mentioned above, the fact that the polyQ region is embedded within a host protein likely affects the in vivo aggregation properties of these chimeras as well. This might explain why aggregation can be observed in case of a polyQ::YFP chimera bearing a similar number of Qs (i.e. 82 versus 72/79 for BlaP chimeras) [32, 41], where the fusion is at YFP’s N-terminus, but not in case of the different BlaP-polyQ chimeras, where the polyQ is embedded within the sequence. Embedded polyQ regions have successfully led to aggregation in *C. elegans* before, however, some of these contained longer polyQ regions (74 Q – 150 Q) longer. In this scenario, this would either mean that aggregation of embedded polyQ requires higher thresholds than terminal polyQ, or that the individual protein context, rather than a general terminal vs. embedded polyQ position might matter. These hypotheses could be verified in follow-up research including N-terminal BlaP-polyQ chimeras, as well as embedded polyQ with longer repeat length.

Sequences immediately adjacent to the polyQ region have shown to be of significant importance for in vivo aggregation (e.g. [40]). Additionally, the ability to modify or interact with these amino acids has been shown to contribute as well: sumoylation facilitates polyQ toxicity in mammalian cells [64]. Mimicking this, research in yeast has shown that pseudosumoylation can be achieved by adding an acidic FLAG-tag [65, 66]. In yet another example of the importance of the flanking region, the absence of proline-rich sequences adjacent to the polyQ region seems to unmask toxicity in yeast as their presence targets the protein to the aggresome [65, 67]. Interestingly, these flanking sequences have not only shown to have cis-regulatory effects, trans effects have been observed as well [66]. Similar observations have been made in *C. elegans* research. Here, interactions between polyQ regions with the mutant ataxin-3 protein-flanking sequences are believed to be crucial for forming aggregates [68]. Together, these findings show that flanking sequences are paramount for polyQ toxicity and aggregation, which could – be it partially or wholly – explain the absence of toxicity of the BlaP-polyQ strains investigated here. One way to address this in the future, would be to express BlaP chimeras with extremely long polyQ repeats (>150 Q), which theoretically might annul the assumedly benefits of the flanking regions regarding solubility and aggregation.

Additionally, *C. elegans* is characterized by a relatively short lifespan. Since the aggregation process of soluble proteins into highly organized amyloid fibrils has been described as a nucleation-dependent polymerization mechanism, it could be that the aggregation process of BlaP-polyQ chimeric proteins takes too long to cause distinct effects in *C. elegans*. This touches upon a more general question regarding the compressibility of ageing in absolute time, and hence of proper modelling of the associated phenotypes in short-living model organisms. Notwithstanding this, *C. elegans* has already proven to be a good model in the study of other aggregation-prone proteins such as amyloid-β or α-synuclein [69–71]. Still, for potentially less toxic proteins this model system might simply be too short-living. Comparative in vivo toxicity studies of these proteins could be conducted in model organisms with a longer lifespan, granting the proteins more time to aggregate. Such studies will demand more time and will probably entail more variation. In an attempt to compensate for the shorter timescale of observation as a consequence of *C. elegans* lifespan, we expressed BlaP197Q72 in a pro-aggregating background, hoping to facilitate the slower nucleation events of amyloid fibril formation [20]. However, also in these animals no aggregation was observed (Fig. 9).

Finally, intracellular conditions (such as pH, temperature and crowding) differ significantly from those used in in vitro studies (i.e. in vitro aggregation studies are carried out in 50 mM phosphate buffer pH 7.5 containing 150 mM NaCl at 37 °C while in vivo experiments occur in a complex cellular environment at 20 °C). Adjustment of these parameters strongly influences the aggregation propensities of the proteins [6, 18]. By relying on an in vivo model, the ability to alter these parameters is limited by cellular and organismal requirements. Although this confers biological significance, it could also impede the aggregation in our models.

### Prospects for BlaP-polyQ chimera aggregation studies in *C. elegans*

*C. elegans* has a good track record as model in the study of protein aggregation [41, 69, 71, 72]. This study shows that *C. elegans’* merits may serve certain studies, but not others, depending on protein identity and context. The inherent toxicity of the studied BlaP-polyQ proteins within this worm’s in vivo context is probably too low to lead to a distinct phenotype. Additionally, all strains used in our experiments were transgenic. Hence, site-specific integration of the transgene in the genome would be desirable since this will reduce the expected variation of the readout drastically. In this way, the copy number and place of integration in the genome can be controlled allowing for a better comparison between the different strains. Bearing in mind that we injected the animals with high concentrations of transgenic target DNA (70 ng/μl), expressed under robust, strong promoters, such site-specific integration of the transgene would likely not enhance aggregation, despite reducing interanimal variation. BlaP chimeras with more aggressive aggregation properties, as discussed above, may still lead to distinct effects, and could therefore benefit from the elaborate *C. elegans* toolbox in their initial in vivo evaluation. One other way to model BlaP-polyQ in *C. elegans*, would be by expressing these proteins in an autophagy-defective background [73], potentially even aggravated by crossing such strains with aggregation-prone backgrounds for seeding. This could yet allow for valuable comparative, highly controllable in vivo studies if interpreted cautiously.

## Conclusion

We have generated a series of transgenic strains expressing BlaP-polyQ proteins bearing polyQ regions of different lengths embedded within two different locations in the BlaP sequence. Despite the verification of proper functional expression at relevant levels of the different proteins, no clear phenotypic consequences were observed. Furthermore, expressing BlaP197Q72 in a pro-aggregating background did not result in accelerated aggregation kinetics. The absence of a toxic in vivo effect is likely a result of several causes. Since *C. elegans* has a relatively short lifespan, the time needed to build up significant aggregation may surpass the worm’s lifespan. Functional protein quality control processes and the differences between in vivo biochemical conditions (such as pH or temperature) and in vitro studies likely all contribute to the observed resistance to toxic aggregation. These observations emphasize the need for proper in vivo evaluation and choice of multiple model systems for comparative purposes and validation of biochemical findings.

## Additional file

Additional file 1: Figure S1. Coomassie briliant blue total protein stain of transgenic strains expressing BlaP_216_Q55-79 and Q82::YFP. **Figure S2.** UPR stress resistance as determined by % survival on day 12 of adulthood on tunicamycin-supplemented medium. **Figure S3.** Thioflavin S staining of potential Q82::YFP and Q86::CFP aggregates. **Figure S4**: Thioflavin S staining of potential BlaP216Q0/55/79 (C/E/G) and BlaP197Q0/58/72 aggregates (D/F/H). **Figure S5.** Verification of the aggregation of BlaP-polyQ proteins by means of SDD-AGE. (DOCX 3398 kb)

## Abbreviations

BlaP: β-lactamase from *Bacillus licheniformis* 749/C
BWM: Body wall muscle cells
CGC: *Caenorhabditis* Genetics Center
GFP: Green fluorescent protein
NGM: Nematode growth medium
PolyQ: Polyglutamine
UPR: Unfolded protein response
YFP: Yellow fluorescent protein
