# Supplementary material for: In vitro aggregating β-lactamase-polyQ chimeras do not induce toxic effects in an in vivo Caenorhabditis elegans model
Source: J Negat Results Biomed. 2017 Aug 22;16:14. doi: 10.1186/s12952-017-0080-5 (PMC5568214; doi:10.1186/s12952-017-0080-5)
Supplement: Additional file 1: Figure S1. — Coomassie briliant blue total protein stain of transgenic strains expressing BlaP216Q55-79 and Q82::YFP. Figure S2. UPR stress resistance as determined by % survival on day 12 of adulthood on tunicamycin-supplemented medium. Figure S3. Thioflavin S staining of potential Q82::YFP and Q86::CFP aggregates. Figure S4: Thioflavin S staining of potential BlaP216Q0/55/79 (C/E/G) and BlaP197Q0/58/72 aggregates (D/F/H). Figure S5. Verification of the aggregation of BlaP-polyQ proteins by means of SDD-AGE. (DOCX 3398 kb) [file 12952_2017_80_MOESM1_ESM.docx]

**Additional file 1**

***In vitro* aggregating β-lactamase-polyQ chimeras do not induce toxic effects in an *in vivo* *Caenorhabditis elegans* model**

Roel Van Assche^1†^, Charline Borghgraef^1†^, Jonathan Vaneyck^2^, Mireille Dumoulin^2^, Liliane Schoofs^1^ and Liesbet Temmerman^1^*

^1^Animal Physiology and Neurobiology, Department of Biology, KU Leuven (University of Leuven), Naamsestraat 59, 3000 Leuven, Belgium

² Enzymology and Protein Folding, Center for Protein Engineering, InBioS, Institute of Chemistry, University of Liège, (Sart-Tilman) 4000 Liège, Belgium

^†^ These authors contributed equally to this work.

*Correspondence: Liesbet Temmerman, Animal Physiology and Neurobiology, KU Leuven, Zoological Institute, Naamsestraat 59, 3000 Leuven, Belgium. e-mail: [liesbet.temmerman@kuleuven.be](mailto:liesbet.temmerman@kuleuven.be)

[roel.vanassche@kuleuven.be](mailto:roel.vanassche@kuleuven.be)

[charline.borghgraef@kuleuven.be](mailto:charline.borghgraef@kuleuven.be)

[jvaneyck@student.ulg.ac.be](mailto:jvaneyck@student.ulg.ac.be)

[mdumoulin@ulg.ac.be](mailto:mdumoulin@ulg.ac.be)

[liliane.schoofs@kuleuven.be](mailto:liliane.schoofs@kuleuven.be)

**Supplemental results and interpretation**

*Tunicamycin stress test indicates no elevated UPR levels in the transgenic strains*

Unfolded protein response levels are known to be elevated as a result of protein misfolding/aggregation [1]. Since only very limited aggregation could be observed (Fig 7), a tunicamycin UPR stress test was conducted to verify whether the expression of BlaP-polyQ chimeras has an influence on UPR stress resistance of *C. elegans*. Unlike the positive control (*xbp-1* mutant) which is unable to induce a heat shock response [2], Q82::YFP and transgenic BlaP-polyQ strains show no increased mortality due to the tunicamycin-induced UPR stress (Additional file 1: Figure S1).

*Thioflavin staining and SDD-AGE could not detect aggregates in positive control strains and/or BlaP-polyQ animals.*

In an attempt to visualize and quantify possible aggregates within our samples, we first relied on thioflavin staining (SFig 3 and 4). While this correctly revealed aggregates in the Aβ control strain, no aggregation could be visualized in any of the polyQ strains, including the clearly aggregating Q82::YFP and Q86::CFP control strains, suggesting thioflavin staining is not suitable for the visualization of these polyQ aggregates (SFig 3 and 4). Therefore, we planned to rely on SDD-AGE assays (SFig 5), which should deliver robust quantification but lack the spatial information that could have been gained via the thioflavin assay. However, while the positive control (Q82::YFP strain) clearly contains aggregates as shown in SFig 3, it was negative for aggregates when evaluated via SDD-AGE (SFig 5). We therefore did not proceed with this analysis.

Several technical reasons may explain the inability to detect aggregates in these strains. For thioflavin experiments, Q82::YFP and Q86::CFP protein aggregates could not be ThS stained either, while aggregates were clearly seen in the fluorescence pattern of GFP/CFP (SFig 3 and 4). Steric hindrance of the fibrils by the still functional YFP/CFP, thus not allowing ThS to interact with the beta sheet structures, likely explains this. This is also supported by the fact that *in vitro* formed fibrils from polyQ proteins very poorly bind ThT [3, unpublished data Thorn D., Huynen C., Pain C & Dumoulin, M.]. For SDD-AGE, it has been reported that the ability to stain aggregates depends on epitope accessibility (hence, the antibody used for detection) [4]. While the antibody used here does bind BlaP-polyQ chimeric fibrils formed *in vitro* [unpublished results Dumoulin, M.], and according to manufacturer’s information is known to bind polyQ in fibrils, we cannot rule out this would be a Q82::YFP-specific issue. At this stage and in combination with the ThS data, this seems unlikely, yet, future research developing a series of antibodies for this purpose would be needed to further address this.

Additional file 1: Figure S1-S5


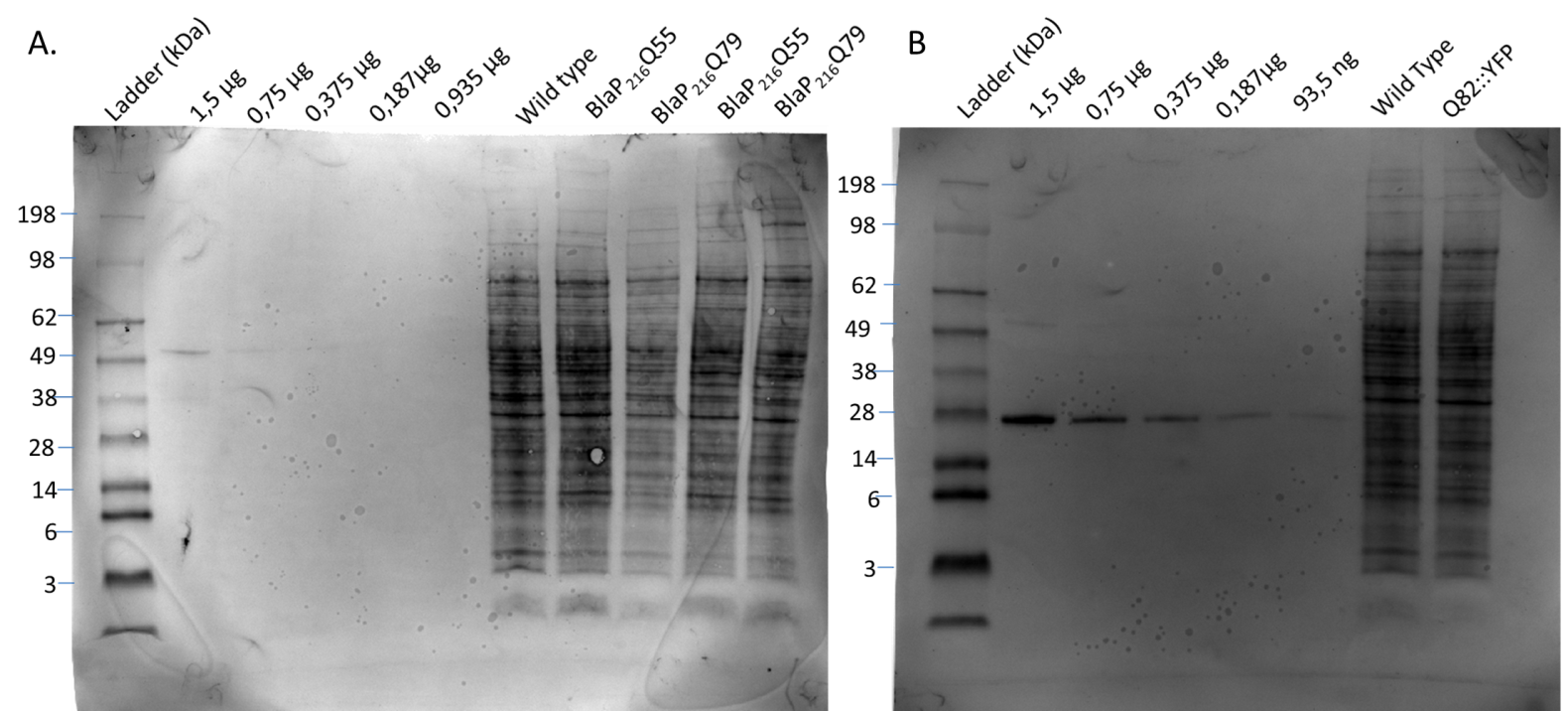


Additional file 1: Figure S1: Coomassie briliant blue total protein stain of transgenic strains expressing BlaP_216_Q55-79 and Q82::YFP. A 2-fold dilution series of pure **(A)** BlaP197Q79 and **(B)** YFP were analysed in order to estimate the order of magnitude of transgenic expression. The total amount of protein was similar in all samples.

**
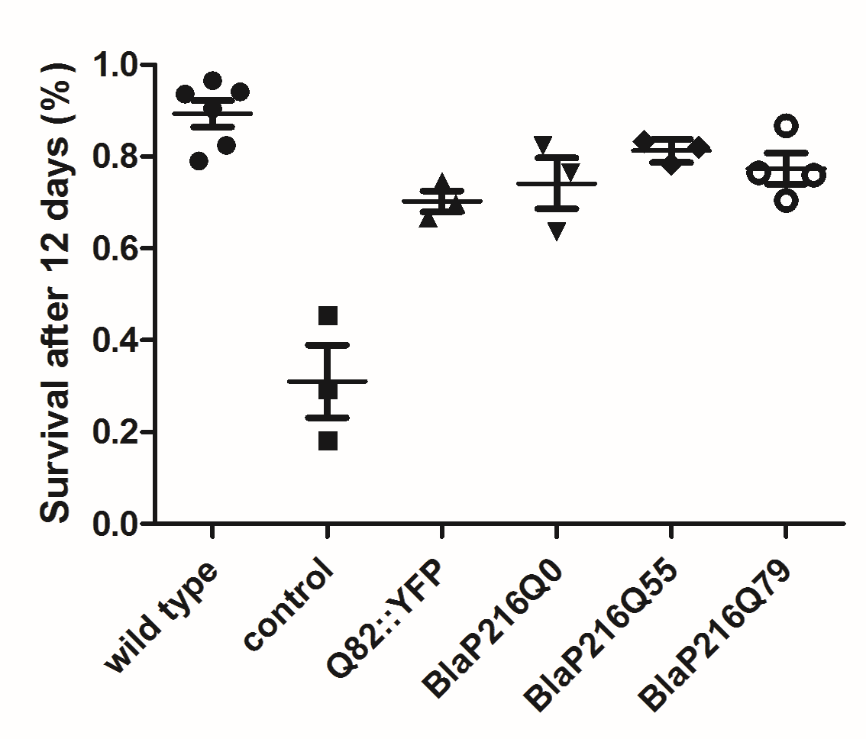
**

Additional file 1: Figure S2**: UPR stress resistance as determined by % survival on
day 12 of adulthood on tunicamycin-supplemented medium.** Each condition represents 3-6 biological replicas with n=20-78 depending on the replica. Error bars indicate standard error of mean. *xbp-1* mutants served as positive control. No significant differences were observed between wild type and polyQ strains.


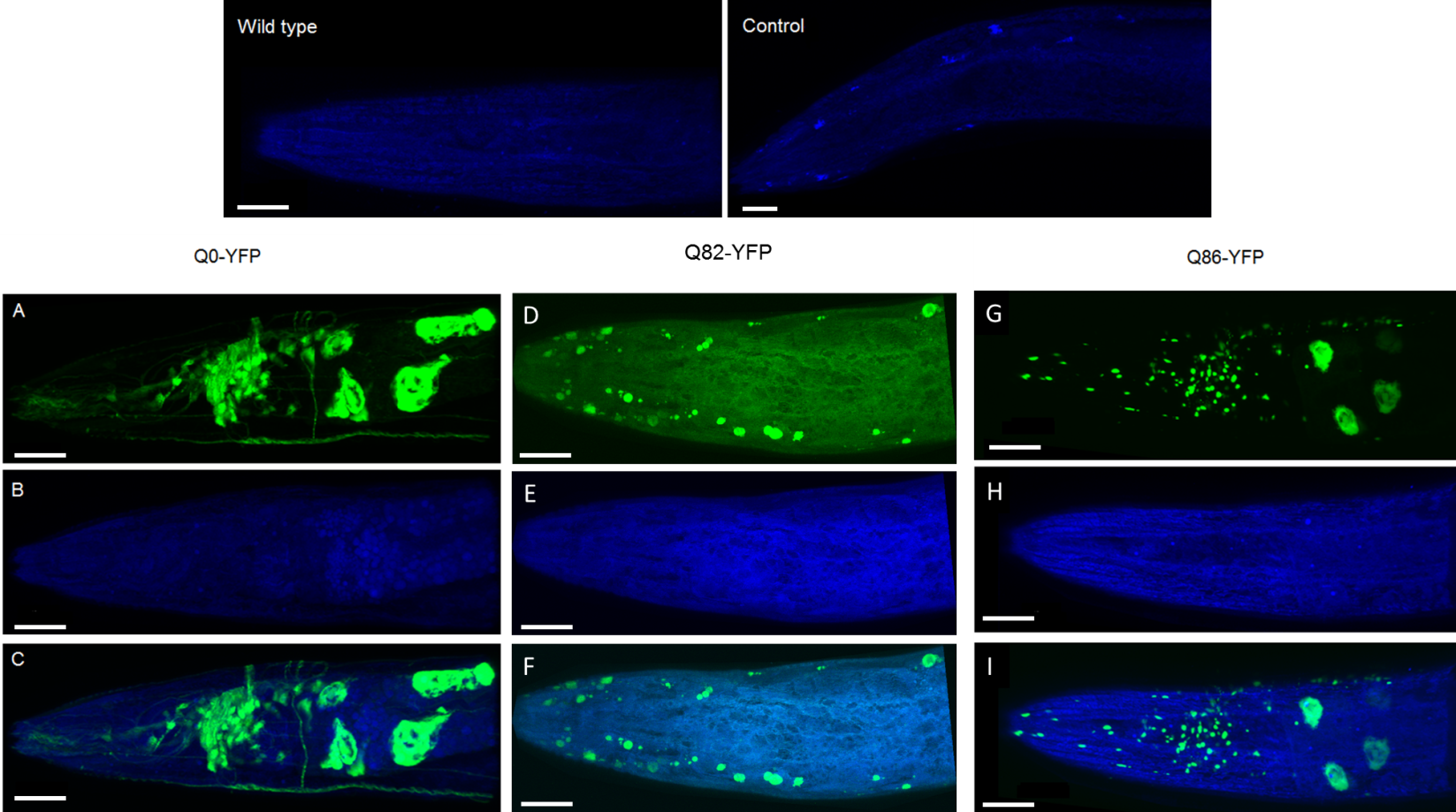


Additional file 1: Figure S3**: Thioflavin S staining of potential Q82::YFP and Q86::CFP aggregates.** Aggregation is only observed in the amyloid-β expressing positive control strain which was used as a positive ThS staining control. **(A-C)** Q0::CFP expressed in the neurons with respectively CFP signal, Thioflavin S signal and an overlay. **(D-F)** Q82::YFP expressed in the body wally muscle cells with respectively YFP signal, Thioflavin S signal and an overlay. **(G-I)** Q86::CFP expressed in the neurons with respectively CFP signal, Thioflavin S signal and an overlay. Although aggregation is visualized by the YFP signal, aggregates could not be stained with Thioflavin S. Scale bars represent 10 µm.

*
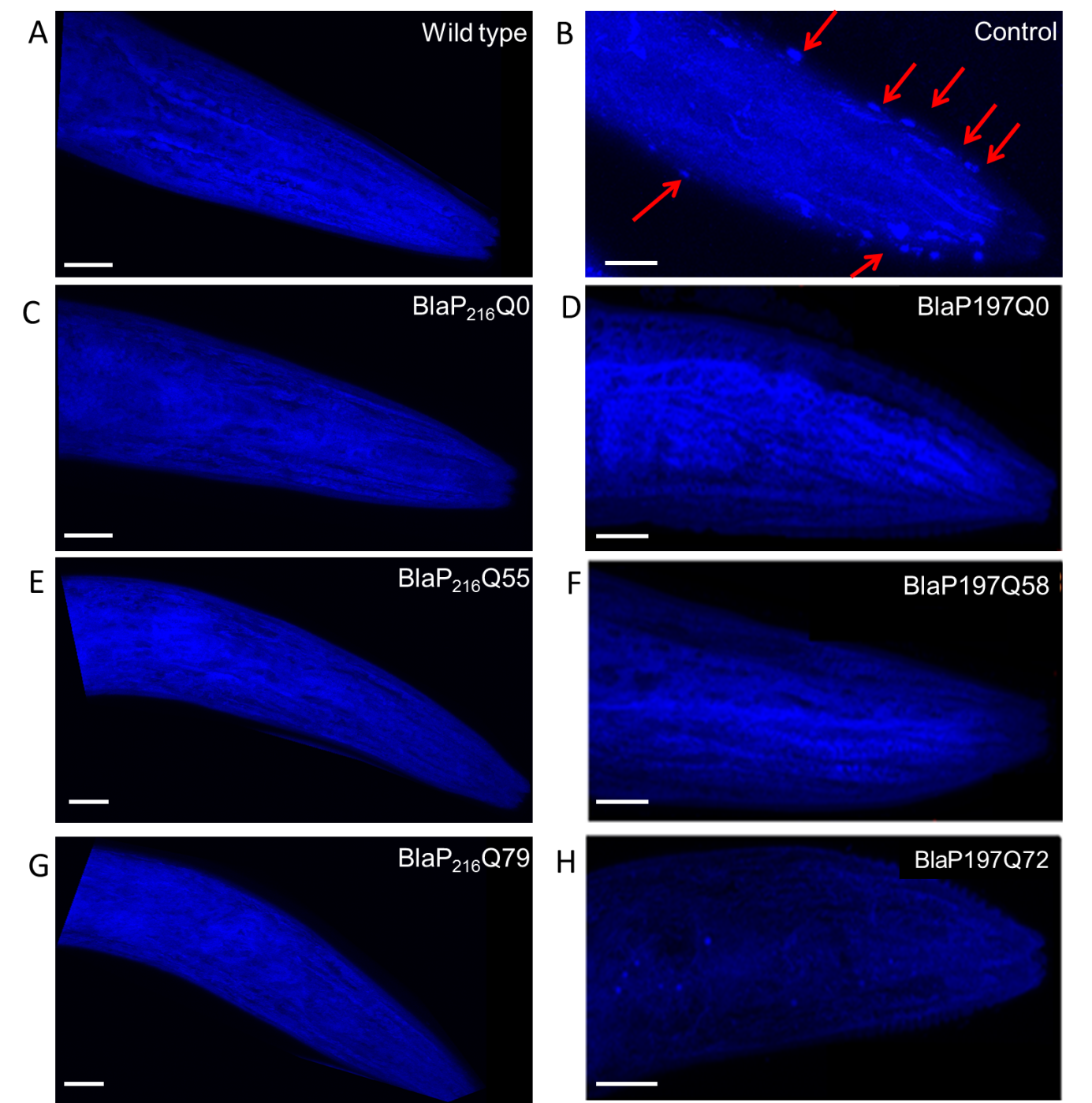
*

Additional file 1: Figure S4**: Thioflavin S staining of potential BlaP216Q0/55/79 (C/E/G) and BlaP197Q0/58/72 aggregates (D/F/H).** All transgenic strains were stained with Thioflavin S. Additionally, wild type **(A)** and a positive control expressing amyloid-β **(B)** were included. Aggregation is only observed in the amyloid-β expressing positive control strain (B). Scale bars represent 10 µm.


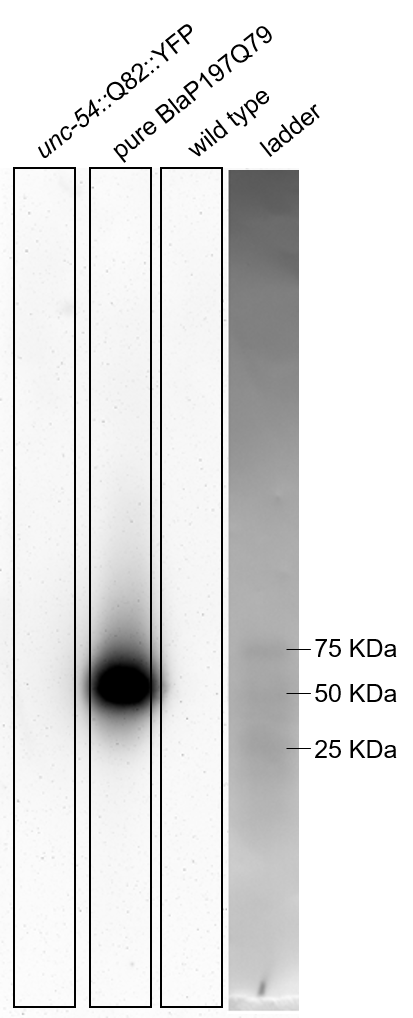


Additional file 1: Figure S5**: Verification of the aggregation of BlaP-polyQ proteins by means of SDD-AGE.** Protein extracts from a total of 50 animals expressing Q82::YFP in body wall muscle cells (*Punc-54*), which functions as positive control, and wild type animals, which functions as a negative control, were loaded (maximum capacity and sample concentration achievable). 74 pmol of pure BlaP197Q79 was loaded as technical control. Compared to the absent signal for the Q82::YFP protein extract, a clear signal is observed for pure BlaP197Q79 monomers, the latter within expectations. Representative blot out of three independent experiments. Vertical lines were added to the image to aid in lane discrimination of the single immunoblot represented in the figure.

**Supplemental methods**

*Tunicamycin unfolded protein response stress assay*

Tunicamycin is a compound known to induce unfolded protein response (UPR) stress [1, 2]. Worms displaying elevated sensitivity to UPR stress will be more susceptible to the toxic effects when cultured on tunicamycin-enriched medium. Synchronized L4 worms were transferred to tunicamycin-containing (10µg/ml) NGM plates and worm survival was measured until day 12 of adulthood. 5-Fluoro-2′-deoxyuridine (856657, Sigma Aldrich, Germany) was added to prevent the development of offspring. Statistical analysis was performed using one-way ANOVA (Graphpad Prism 5, GraphPad Software, USA). *P* values < 0.05 were considered significant.

*Thioflavin staining*

Thioflavin-S (ThS) staining [5] was performed to visualize protein aggregates in *C. elegans*. Synchronized worms were first fixed for 24 h at 4 °C in 4% paraformaldehyde/phosphate buffered saline (PBS; 125 mM NaCl, 16 mM Na_2_HPO_4_, 8 mM NaH_2_PO_4_.2H_2_O, pH = 7.4). Permeabilization of the animals was achieved by incubating them in 5% mercaptoethanol, 1% Triton X-100, 125 mM Tris, pH 7.4 for 24 h at 37 °C. Staining of the permeabilized worms was established by adding 0.125% ThS in 50% ethanol to the worms for 2 min. Next, destaining was performed in 50% ethanol. Worms were mounted on a fresh 2% agarose pad for confocal microscopy (Olympus Fluoview FV1000 (IX81)). Whole-diameter Z-stack images were created to visualize protein aggregates and Confocal Z-stack projections were exported through Imaris 7.2 (Olympus, Germany).

*SDD-AGE*

In order to visualise aggregates, a semi-denaturating detergent agarose gel electrophoresis (SDD-AGE) was performed. Samples were collected as described before (Western blot).

100 mL of a 1.5% agarose solution was prepared in 1X TAE. After boiling, SDS was added to 0.1% (w/v). The solution was poured in a 12 cm x 12 cm gel casting tray, air bubbles were removed and the gel was allowed to solidify. 30 µl of each sample were loaded and the samples were run in 1X TAE buffer containing 0.1% SDS at low voltage (about 3 V/cm gel length) for approximately 6 hours at 4°C. Meanwhile, about 30 pieces of GB002 blotting paper (Macherey-Nagel) of 12 cm x 12 cm were cut, 28 pieces of blotting paper were placed on top of each other and a prewetted piece of blotting paper was placed on top of this stack. A piece of 0.43 µm nitrocellulose was cut and prewetted after which it was carefully placed on top of the stack. The gel was removed from the casting tray, rinsed with water and placed on top of the stack. This was topped off with another piece of prewetted blotting paper and a prewetted wick, which was submerged in 1X TBS at both ends. To ensure thorough contact between all layers and to remove air bubbles, a pipette was firmly rolled across the top of the stack. Next, the transfer was allowed to proceed overnight. After transfer, the nitrocellulose membrane was processed as described for Western blotting.

**References**

1. Kim HM, Do C-H, Lee DH. Taurine reduces ER stress in C. elegans. J Biomed Sci. 2010;17 Suppl 1 Suppl 1:S26.

2. Bischof LJ, Kao C-Y, Los FCO, Gonzalez MR, Shen Z, Briggs SP, et al. Activation of the unfolded protein response is required for defenses against bacterial pore-forming toxin in vivo. PLoS Pathog. 2008;4:e1000176.

3. Scarafone N, Pain C, Fratamico A, Gaspard G, Yilmaz N, Filée P, et al. Amyloid-Like Fibril Formation by PolyQ Proteins: A Critical Balance between the PolyQ Length and the Constraints Imposed by the Host Protein. PLoS One. 2012;7:e31253.

4. Halfmann R, Lindquist S. Screening for Amyloid Aggregation by Semi-Denaturing Detergent-Agarose Gel Electrophoresis. 2008;:e838. doi:doi:10.3791/838.

5. Link CD. Expression of human beta-amyloid peptide in transgenic Caenorhabditis elegans. Proc Natl Acad Sci U S A. 1995;92:9368–72.
